# Dorsal Striatum and Its Limbic Connectivity Mediate Abnormal Anticipatory Reward Processing in Obesity

**DOI:** 10.1371/journal.pone.0031089

**Published:** 2012-02-03

**Authors:** Lauri Nummenmaa, Jussi Hirvonen, Jarna C. Hannukainen, Heidi Immonen, Markus M. Lindroos, Paulina Salminen, Pirjo Nuutila

**Affiliations:** 1 Brain Research Unit, Olli Viktor Lounasmaa Laboratory, Aalto University School of Science, Espoo, Finland; 2 Department of Biomedical Engineering and Computational Science, Aalto University School of Science, Espoo, Finland; 3 Turku Positron Emission Tomography (PET), Centre, Turku, Finland; 4 Department of Medicine, University of Turku, Turku, Finland; 5 Department of Surgery, University of Turku, Turku, Finland; Louisiana State University Health Sciences Center, United States of America

## Abstract

Obesity is characterized by an imbalance in the brain circuits promoting reward seeking and those governing cognitive control. Here we show that the dorsal caudate nucleus and its connections with amygdala, insula and prefrontal cortex contribute to abnormal reward processing in obesity. We measured regional brain glucose uptake in morbidly obese (*n* = 19) and normal weighted (*n* = 16) subjects with 2-[18F]fluoro-2-deoxyglucose ([^18^F]FDG) positron emission tomography (PET) during euglycemic hyperinsulinemia and with functional magnetic resonance imaging (fMRI) while anticipatory food reward was induced by repeated presentations of appetizing and bland food pictures. First, we found that glucose uptake rate in the dorsal caudate nucleus was higher in obese than in normal-weight subjects. Second, obese subjects showed increased hemodynamic responses in the caudate nucleus while viewing appetizing versus bland foods in fMRI. The caudate also showed elevated task-related functional connectivity with amygdala and insula in the obese versus normal-weight subjects. Finally, obese subjects had smaller responses to appetizing versus bland foods in the dorsolateral and orbitofrontal cortices than did normal-weight subjects, and failure to activate the dorsolateral prefrontal cortex was correlated with high glucose metabolism in the dorsal caudate nucleus. These findings suggest that enhanced sensitivity to external food cues in obesity may involve abnormal stimulus-response learning and incentive motivation subserved by the dorsal caudate nucleus, which in turn may be due to abnormally high input from the amygdala and insula and dysfunctional inhibitory control by the frontal cortical regions. These functional changes in the responsiveness and interconnectivity of the reward circuit could be a critical mechanism to explain overeating in obesity.

## Introduction

In most western countries the annual increase in the prevalence and the severity of obesity is currently substantial [1]. Unrestricted availability of palatable foods is the most obvious environmental factor that promotes obesity [2], and genes promoting rapid intake of energy via high sugar and fat intake under conditions of food scarcity have become a liability in the modern societies where high-caloric food is ubiquitously available. To fight the current obesity epidemic it is thus imperative to understand which factors determine whether food consumption is pursued or restrained. Eating provides nutrients but is also highly reinforcing, because it induces intense feelings of pleasure and reward. Comparative studies have established that an interconnected *reward circuit* comprising of subcortical (amygdala, hypothalamus, striatum) and frontocortical (motor, premotor, orbital and medial prefrontal) areas plays a key role in guiding appetitive behaviours [3], [4], [5]. Functional imaging studies in humans have further shown that subcomponents of the reward circuit contribute to processing of external food cues such as pictures of foods [6], [7], [8], [9], and dysfunctions of the reward circuit have also been associated with both obesity and drug addiction. [2], [10], [11], [12], [13], [14]. In the present study we show how tonic activity, regional responses as well as interconnectivity of the reward circuit may be the critical mechanisms explaining overeating and obesity.

Palatable foods carry strong motivational power. Mere sight of a delicious cake or the smell of our favourite food may elicit a strong urge for eating right now, and exposure to such cues may override physiological satiety signals and trigger food consumption [15]. Overeating thus likely depends on the balance between the reward circuit and networks that inhibit reward seeking, such as the dorsolateral prefrontal cortices [16], [17], [18]. The extant literature from imaging studies in humans suggests that obesity is characterized by an imbalance in these systems, in that the reward circuit it is overactive to reward anticipation in obesity and that inhibitory networks may fail to exert control over the the reward circuit [2], [10], [11], [12], [13], [14], [19]. There are large individual differences in the reward circuit's responsiveness towards foods, and this may be a critical factor contributing to overeating and obesity [2]. The personality trait reward drive is positively associated with food cravings and body weight [20], and fMRI studies have revealed that it also predicts ventral striatum's responses to appetizing food pictures in normal-weight individuals [21]. Similarly, self-reported sensitivity to external food cues is positively correlated with the interconnectivity of the reward circuit [22]. In line with these findings, fMRI studies have confirmed that the reward circuit of obese individuals is hypersensitive to the mere sight of foods. Obese individuals show elevated responses to food pictures in amygdala, caudate nucleus and anterior cingulate cortex [10], [19], and it has been proposed that this hyperactivity of the dopaminergic reward circuit may render obese individuals prone to overeating. PET studies have further demonstrated dopaminergic commonalities in the mechanisms of drug abuse and excessive food intake, suggesting that at least in some cases obesity might be characterized as a ‘food addiction’. Dopaminergic reward pathways in the midbrain modulate both food and drug consumption [23] particularly by means of creating sensations of food and drug craving [24], and both drugs and food exert their reinforcing effects by increasing dopamine in limbic regions. Patients with addictive disorders show tonically lower baseline D_2_ receptor (D_2_R) density in the striatum, and blunted dopamine release following the administration of the drug of abuse. Similar to drugs of abuse, food consumption is associated with dopamine release in the dorsal striatum in healthy subjects, and the amount of dopamine released is correlated positively with ratings of food pleasantness [12]. Similar to patients with addictive disorders, obese subjects have lower baseline striatal D_2_R density, which is directionally proportional to BMI [11].

Although altered sensitivity of the reward circuit may be a critical factor explaining obesity, it remains elusive how exactly the reward circuitry contributes to food-related anticipatory reward functions in obese individuals. First, previous demonstrations of elevated reward circuit responses to foods in normal-weight and obese subjects [10], [19] have not addressed differences in the tonic baseline activity of the reward circuit in the brain. Tonically low glucose metabolism in the prefrontal cortex predicts low striatal dopamine D_2_ receptor density - a hallmark of dysregulated reward circuit - in obese subjects [17]. However, whether tonic activity of the neural networks that process anticipatory reward predicts functional responses to external food cues is unknown. Second, only a handful of studies have taken a systems-level approach for testing whether obesity would alter the functional *connectivity* of the reward circuit. While a recent imaging study in healthy humans demonstrated that connectivity within the human reward circuit is dependent on individual sensitivity to external food cues [22], another involving obese and normal-weight individuals suggested that obesity is specifically associated with deficient functional connectivity from amygdala to the orbitofrontal cortex, (OFC) and heightened connectivity from the OFC to ventral striatum [25]. However, the exact neural mechanisms underlying these functional changes remain unknown.

In this study we applied multimodal brain imaging by combining [^18^F]FDG PET with an fMRI experiment involving anticipatory reward induced by presentation of appetizing and bland food pictures. Note that although no rewards were actually delivered to the participants, we use the term ‘anticipatory reward’ for the sake of conciseness, as seeing highly rewarding targets such as foods reliably induces reward anticipation responses in the ventral striatum, even when no rewards are actually delivered [21]. It has been established that glucose utilization is tightly associated with spiking frequency [26], hence the glucose metabolism rates can be used to measure tonic baseline activation of the brain during rest. By using primed hyperinsulinemic clamp [27] during the PET scan, we were able to compare obese and normal-weight individuals' brain glucose metabolism in a situation where the body is in a satiated state in terms of insulin signaling. The fMRI experiment enabled us to compare whether obese and normal-weight individuals differ with respect to both regional brain responses and effective connectivity of the reward circuit during viewing of appetizing vs. bland foods. Finally, combining the PET and fMRI data enabled us to use the regional glucose metabolic rates (GMRs) derived in the PET scan to predict brain responses to appetizing foods in the fMRI experiment.

## Materials and Methods

### Participants

The Ethical Committee of the Hospital District of South-Western Finland approved the study protocol and all participants signed ethical committee-approved, informed consent forms. The study was conducted in accordance with the Declaration of Helsinki. Table 1 presents a summary of the participants. The obese group consisted of nineteen neurologically intact morbidly obese subjects (*M*_BMI_ = 43.87, *SD*_BMI_ = 6.60). Five of them used oral antidiabetic medication and were excluded from the PET studies. Sixteen neurologically intact normal-weight volunteer subjects served as controls (*M*_BMI_ = 24.10, *SD*_BMI_ = 2.07) and were matched with the patients with respect to age, height, and indices of hypertension (i.e. blood pressure). Eating disorders, severe mental disorders and substance abuse were exclusion criteria for all participants. One normal-weight subject **was excluded from the fMRI data analyses due to excessive head motion.**

**Table 1:** Characteristics of the participants.

	Obese subjects (n = 19)		Normal-weight subjects (n = 16)		
	*M*	*SD*	*M*	*SD*	*p*
Age (years)	45.74	9.60	47.75	10.44	ns.
Weight (kg)	123.03	11.20	71.43	12.00	<.001
Height (cm)	167.47	6.46	171.59	10.34	ns.
BMI	43.87	3.74	24.10	2.07	<.001
Percentage of fat*	48.27	6.60	29.37	6.37	<.001
Systolic blood pressure (mmHg)	133.94	14.06	125.71	12.52	ns.
Diastolic blood pressure (mmHg)	85.83	8.21	80.43	8.59	ns.
Appetizing foods rating#	6.16	1.13	5.97	1.08	ns.
Bland foods rating#	5.18	1.59	4.68	1.65	ns.
Cars rating#	5.55	1.51	4.67	2.02	ns.
VAS hunger rating	28.20	24.87	36.75	28.19	ns.

Last column indicates significant between-groups differences in a two-sample *t* test.

*Note.*

*Measured using the bioelectrical impedance analysis (BIA) technique with an Omron device.

# Measured with the Self-assesment Manikin using a scale ranging from 1 to 9.

### Behavioural measurements

Prior to the experiment, participants rated their feeling of hunger using a visual analogue scale. After the fMRI experiment, the participants rated the valence (pleasantness versus unpleasantness) of the experimental stimuli on a computer using the Self-assessment Manikin [28] with a scale ranging from 1 (unpleasant) to 9 (pleasant).

### PET acquisition and analyses

The studies were performed after 12 hours fasting. Subjects refrained from caffeine-containing drinks and from smoking 24 hours before PET studies. Any kind of strenuous physical activity was prohibited from the preceding evening. Two catheters were inserted into antecubital veins, one for saline, insulin and glucose infusions and injection of radiotracer [^18^F] FDG, and another into the opposite warmed arm for sampling of arterialized blood. The euglycemic hyperinsulinemic clamp technique was used as previously described [27]. The rate of insulin infusion was 1 mU · kg^−1^ · min^−1^ (Actrapid, Novo Nordisk, Copenhagen, Denmark). During hyperinsulinemia, euglycemia was maintained by infusing 20% glucose intravenously. The rate of glucose infusion was adjusted according to plasma glucose concentrations measured every 5–10 min from arterialized blood. At the time point 100+−10 minutes of euglycemic hyperinsulinemic clamp, [^18^F]FDG (189±9 MBq) was injected intravenously over 40 second and the dynamic brain scan for 40 min (frames; 4 • 30 s, 3 • 60 s, 7 • 300 s) started. During the scan arterial blood samples were drawn for radioactivity analysis. A GE Advance PET scanner (General Electric Medical Systems, Milwaukee, WI, USA) with resolution of 4.25 mm was used for PET studies as previously described [29], [30]. [^18^F]FDG was synthesized as previously described [31]. Plasma radioactivity was measured with an automatic gamma counter (Wizard 1480 3″, Wallac, Turku, Finland).

Cerebral glucose uptake rate was measured for each voxel separately from dynamic PET scans as described previously [29], [30], except that a lumped constant of 0.8 was used [32]. Normalization and statistical analyses of the parametric glucose metabolism images were carried out with SPM 5 software (www.fil.ion.ucl.ac.uk/spm/). Parametric images were normalized into an in-house glucose metabolism template in MNI space using linear and nonlinear transformations, and smoothed with a Gaussian kernel of FWHM 10-mm. Simple t-contrasts for the normalized parametric images were used to analyze group differences in glucose metabolism. The statistical threshold was set at p<.001, uncorrected, with a minimum cluster size of 100 contiguous voxels. For small volume corrections (SVC) in the PET data, anatomically defined *a priori* regions of interest within the reward system (caudate nucleus, amygdala, thalamus, insula and orbitofrontal cortex) were defined using the WFU pickatlas [33] and AAL [34] atlas.

### Experimental Design for fMRI

**Figure 1:**
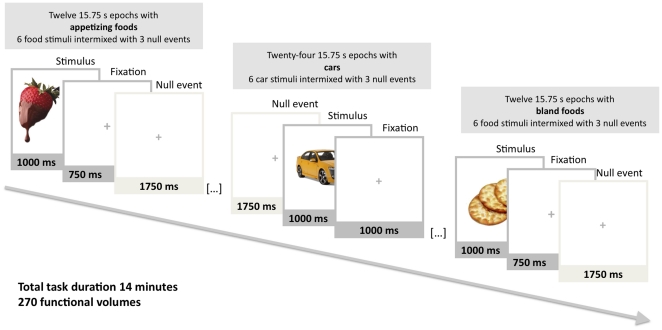
Experimental design for fMRI and examples of the stimuli used. The participants viewed alternating 15.75 epochs of appetizing foods, cars, and bland foods. Each epoch consisted of six experimental stimuli pseudorandomly intermixed with three null events.

Stimuli and design are summarized in Figure 1. The stimuli were digitized full-color photographs of appetizing foods (e.g. chocolate, pizza, steak), bland foods (e.g. lentils, cabbage, crackers) and cars matched with respect to low-level visual features such as mean luminosity, RMS contrast and global energy. An independent sample of 29 healthy volunteers rated the valence (unpleasantness versus pleasantness) of the stimuli with the SAM. Analysis of the valence ratings (*M*_appetizing_ = 6.64, *M*_bland_ = 3.93, *M*_cars_ = 4.41) established that the appetizing foods were rated as more pleasant than the bland foods, *t*(28) = 10.97, *p*<.001, and cars, *t*(28) = 7.52, *p*<.001, but there were no differences in the pleasantness of the bland foods and cars, *t*(28) = 1.19.

While being scanned the subjects viewed alternating 15.75-second epochs containing six stimuli from one category (appetizing foods, bland foods or cars) intermixed with three null events. In order to study implicit processing of the food images, we used brief stimulus display durations and a behavioural task that was unrelated to the hedonic value of the stimuli: A single trial comprised a 1000 ms presentation of a stimulus image followed by a low contrast central cross (750 ms). Null events comprised a 1750 ms presentation of a low-contrast cross. The food and car stimuli were displaced slightly to the left or to the right of the screen, and the participants were instructed to press the left or right button according to which side the stimulus was presented. On null trials no response was demanded. The order of the stimuli during each epoch was pseudo-randomized with respect to trial type (stimulus or null), such that no more than three consecutive trials were of the same type. This pseudo-randomization enhanced design efficiency while preserving the unpredictability of stimulus onsets in naïve participants [35]. Visual field of the stimuli was randomized and fully counterbalanced. Altogether there were a total of 72 appetizing food trials (in 12 epochs), 72 bland food trials (in 12 epochs) and 144 car trials (in 24 epochs). To maximize the power of the design and to prevent carryover effects of viewing appetizing foods, the order of the stimulus epochs was fixed in such way that car stimulus epoch was always presented between the appetizing and bland stimulus epochs. The starting epoch of the task was counterbalanced across participants. The total task duration was 14 minutes. Participants practiced the task outside the scanner prior to starting the fMRI experiment.

### fMRI Acquisition and Analysis

Scanning sessions took place around morning or early noon (9 a.m.–2 p.m.) Participants were instructed to refrain from eating and drink only water for at least three hours prior to scanning. MR imaging was performed with Philips Gyroscan Intera 1.5 T CV Nova Dual scanner at Turku PET centre. High-resolution anatomical images (1 mm^3^ resolution) were acquired using a T1-weighted sequence (TR 25 ms, TE 4.6 ms, flip angle 30°, scan time 376 s). Whole-brain functional data were acquired with echo-planar imaging (EPI) sequence, sensitive to the blood-oxygen-level-dependent (BOLD) signal contrast (TR = 3000 ms, TE = 50 ms, 90° flip angle, 192 mm FOV, 64×64 matrix, 62.5 kHz bandwidth, 4.0 mm slice thickness, 0.5 mm gap between slices, 30 interleaved slices acquired in ascending order). A total of 270 functional volumes were acquired, and the first 5 volumes were discarded to allow for equilibration effects. Data were preprocessed and analyzed using SPM5 software (www.fil.ion.ucl.ac.uk/spm/). The EPI images were sinc interpolated in time to correct for slice time differences and realigned to the first scan by rigid body transformations to correct for head movements. EPI and structural images were coregistered and normalized to the T1 standard template in MNI space (Montreal Neurological Institute (MNI) – International Consortium for Brain mapping) using linear and non-linear transformations, and smoothed with a Gaussian kernel of FWHM 8-mm.

### Analysis of regional effects

A whole-brain random effects model was implemented using a two-stage process (first and second level). This random-effects analysis assessed effects on the basis of inter-subject variance and thus allowed inferences about the population that the participants were drawn from. For each participant, we used a GLM to assess regional effects of task parameters on BOLD indices of activation. The model included three experimental conditions (appetizing foods, bland foods and cars) and effects of no interest (realignment parameters) to account for motion-related variance. Low-frequency signal drift was removed using a high-pass filter (cutoff 128 sec) and AR(1) modeling of temporal autocorrelations was applied. The individual contrast images were generated using the contrast appetizing – bland foods, as well as for the main effect of foods (i.e. appetizing and bland foods against other effects of interest). The second level analysis used these contrast images in a new GLM, and generated statistical images, that is, SPM-t maps. With balanced designs at first level (i.e. similar events for each subject, in similar numbers) this second level analysis closely approximates a true mixed effects design, with both within and between subject variance. Initial analysis revealed that none of the second-level between-groups contrasts was significant when strict false discovery rate (FDR) correction at *p*<.05 was applied. Accordingly, the statistical threshold was set at p<.005, uncorrected, with a minimum cluster size of 20 contiguous voxels for the between-group comparisons.

### Psychophysiological interaction (PPI) in the general linear model (GLM)

The physiological connectivity between two brain regions can vary as a function of the psychological context [36] known as a Psychophysiological Interaction (PPI). PPIs can be identified by general linear models sensitive to contextual modulation of task-related covariance. In contrast with dynamic casual modeling or structural equation modeling of network connectivity, PPIs do not require a specified anatomical model. Rather, one starts with a ‘source’ region and identifies any other ‘target’ voxels/clusters in the brain with which that source has context-dependent connectivity. Target regions need not correlate with the task or context alone, but the interactions between these factors. Significant PPIs do not in themselves indicate the direction or neurochemistry of causal influences between source and target regions, nor whether the connectivity is mediated by mono- or poly-synaptic connections, nor changes in structural neuroplasticity from epoch to epoch. However, they *do* indicate interactions between regional systems, and the results of PPIs accord with other connectivity methods such as dynamic causal modelling [37].

Right caudate nucleus was used as the source region for the connectivity analyses for the appetizing minus bland foods contrast. Global maximum (2, 8, 4) for this region in the second-level obese versus normal-weight contrast in the PET data analyses (see below) was used to derive a statistically independent estimate for the center of the source region; this effectively guarded against ‘double dipping’ in source region selection [38], and enabled theoretically plausible integration of the PET and fMRI data. A spherical ROI with a 10 mm radius was generated at this location. The time-series for each participant was computed by using the first eigenvariate from all voxel time series in the ROI. This BOLD time series was deconvolved to estimate a ‘neuronal time series’ for this region using the PPI-deconvolution parameter defaults in SPM5 [39]. The psychophysiological interaction term (PPI regressor) was calculated as the element-by-element product of the ROI neuronal time series and a vector coding for the main effect of task (i.e. 1 for appetizing foods, −1 for bland foods). This product was then re-convolved by the canonical hemodynamic response function (hrf). The model also included the main effects of task convolved by the hrf, the ‘neuronal time series’ for each ‘source’ and the movement regressors as effects of no interest. Subject-wise PPI models [36] were run, and contrast images were generated for positive and negative PPIs. This whole-brain analysis identified regions have greater or lesser change in connectivity with the source region according to context (i.e., appetizing versus bland foods). The contrast images were then entered into second-level GLM analyses for contrasts of interest, and SPM *t*-maps generated using Gaussian Random Field theory to make statistical inferences.

## Results

### Behavioral measurements

The stimulus valence ratings were analyzed with a 3 (stimulus: appetizing food vs. bland food vs. cars)×2 (group: obese vs. normal-weight) mixed ANOVA. This revealed that the valence ratings differed significantly across stimulus categories, *F*(2,60) = 6.01, *p* = .004, η_p_^2^ = .17, but were similar across obese and normal-weight groups (*F* = 1.46). Multiple comparisons with Bonferronni corrections revealed that participants rated appetizing foods as more pleasant than bland foods, *t*(31) = 4.67, *p*<.001, or cars, *t*(31) = 2.76, *p* = .01, but did not rate bland foods as more pleasant than cars, *t*(31) = .41. Hunger ratings were also equal across patient and control groups (*p*>.05).

### Brain glucose metabolism

**Figure 2:**
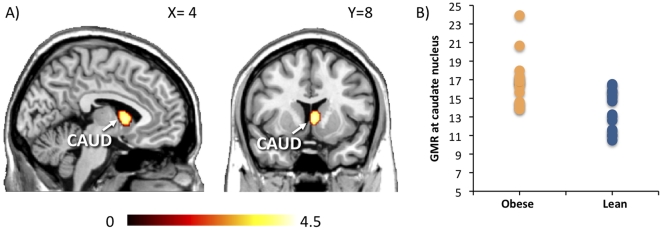
PET scans with 2-[18F] FDG during euglycemic hyperinsulinemia show that glucose metabolic rate (GMR, µmol/100 g*min) in the right caudate nucleus (*X* = 4, *Y* = 8, *Z* = 4) was significantly higher in obese rather than in normal-weight subjects (*p*<.05, SVC). Panel *A* shows the statistical parametric map of the between-group effect, panel *B* shows the subject-wise GMR values in the caudate nucleus.

Obese subjects had significantly higher glucose metabolism in the right caudate nucleus than did normal-weight subjects (*X* = 4, *Y* = 8, *Z* = 4, *T* = 3.97, *p* = .03, SVC) (Figure 2), but not in any other *a priori* region of interest (amygdala, thalamus, insula, or orbitofrontal cortex).

### Regional effects in fMRI

**Figure 3:**
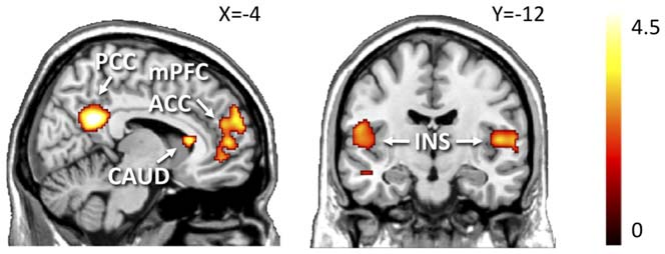
Brain regions showing increased response to appetizing vs. bland foods across all subjects. Appetizing foods increased activity in anterior (ACC) and posterior cingulate cortex (PCC), medial prefrontal cortex (mPFC), right caudate nucleus (CAUD) and bilateral insula (INS). The data are plotted at *p*<.005, uncorrected for visual inspection.

Across all subjects, contrasting appetizing versus bland foods resulted in robust activation of the reward circuit. Activation foci were observed in the medial prefrontal cortex, anterior cingulate gyrus, right ventral striatum, bilateral posterior insula, and posterior cingulate gyrus and precuneus (Figure 3, Table 2). However, between-groups analysis revealed that coding for anticipatory reward was contingent on obesity. Responses to all foods (appetizing *and* bland) were higher in obese than in normal-weight subjects in the left amygdala, hippocampus, posterior cingulate cortex and fusiform gyrus, as well as the right somatosensory cortex. However, responses were lower in obese than in normal-weight subjects in the left superior frontal gyrus. Table 3 presents a summary of these activation foci.

**Table 2:** Brain regions showing increased response to appetizing versus bland foods across all subjects, p<.05 (FDR corrected).

Region	Laterality	X	Y	Z	T
Cerebellum	R	26	−48	−20	6.78
Inferior occipital gyrus	L	−16	−100	−6	6.58
Posterior cingulate gyrus, Precuneus, Cuneus	R	6	−50	24	6.46
Insula, Superior temporal gyrus	R	50	−6	4	6.23
Inferior occipital gyrus	R	28	−96	−12	6.07
Superior frontal gyrus, Anterior Cingulate Gyrus	L	−10	60	18	5.66
Middle temporal gyrus	R	44	−40	2	5.04
Superior temporal gyrus	L	−48	−28	14	4.92
Insula, Superior temporal gyrus	L	−52	−14	12	4.78
Precuneus, Superior parietal lobule	R	14	−48	62	4.77
Postcentral gyrus, Somatosensory cortex	L	−44	−26	54	4.69
Insula, Putamen	L	−34	−20	2	4.38
Caudate nucleus	R	10	22	6	4.35
Supplementary Motor Area	R	8	−10	66	4.1
Postcentral gyrus	L	−24	−42	56	3.98
Middle and posterior cingulate gyrus	R	2	−28	34	3.87
Middle frontal gyrus	L	−24	26	40	3.86
Middle temporal gyrus	L	−46	−68	20	3.84
Fusiform gyrus	L	−26	−52	−16	3.83
Middle cingulate gyrus	R	2	−14	38	3.72

**Table 3:** Between-group (obese vs. normal-weight and normal-weight vs. obese) differences in cerebral responses to all (appetizing and bland) food pictures, p<.005 (unc.).

Region	Laterality	x	y	z	T
Larger response to foods in obese versus normal-weight individuals					
Amygdala/Hippocampus	L	−30	−10	−26	3.89
Posterior cingulate cortex	L	8	−38	18	3.84
Supramarginal gyrus (somatosensory cortex)	R	56	−16	30	3.81
Fusiform gyrus	L	−40	−58	−10	3.76
Larger response to foods in normal-weight versus obese individuals					
Superior Frontal Gyrus	L	−42	4	10	5.21

**Figure 4:**
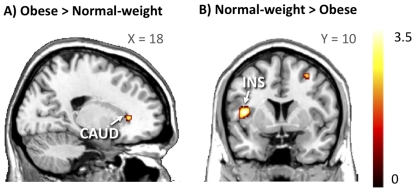
Differential BOLD responses to appetizing and bland foods in normal-weight and obese subjects in the caudate nucleus and anterior insula. Brain responses to appetizing vs. bland foods were larger in the head of the right caudate nucleus (CAUD) of the obese patients, whereas responses to appetizing vs. bland foods were larger in the right anterior insula (INS) of the normal-weight individuals. The data are plotted at *p*<.005, uncorrected for visual inspection.

Next, we asked whether obese subjects would show greater functional responses specifically to appetizing rather than bland foods. To that end, we applied an interaction analysis between group (obese, normal-weight) and food type (appetizing, bland). Consistent with the prediction that obesity would be associated with hyperactivity in the reward circuit, the response to appetizing versus bland foods in the right caudate nucleus was greater in obese than in normal-weight individuals (Figure 4a, Table 4). In contrast, obese subjects had smaller functional responses to appetizing versus bland foods than did normal-weight subjects in the left insula, lateral frontal cortex, superior parietal lobule, right orbitofrontal cortex and superior temporal gyrus (Figure 4b, Table 4). Thus, obese subjects appeared to have an imbalance in regional functional responses to anticipated food reward: greater responses in the caudate nucleus and smaller responses in several frontal cortical regions.

**Table 4:** Between-group (obese vs. normal-weight and normal-weight vs. obese) differences in cerebral responses to appetizing versus bland foods, p<.005 (unc.).

Region	Laterality	x	y	z	T
Larger response to appetizing versus bland foods in obese versus normal-weight individuals					
Caudate	R	18	30	4	4.77
Larger response to appetizing versus bland foods in normal-weight versus obese individuals					
Insula	L	−42	4	10	5.21
Orbitofrontal cortex	R	20	34	−8	4.83
Orbitofrontal cortex (IFG)	R	38	32	−8	4.28
Temporal pole	R	46	12	−26	4.06
Superior Temporal Gyrys	R	52	−38	18	3.99
Superior frontal sulcus	R	20	40	38	3.81
Supramarginal Gyrus	R	38	−38	38	3.65
Inferior Frontal Gyrus	L	−38	34	10	3.54
Middle Frontal Gyrus	R	30	6	54	3.53
Middle Occipital Gyrus	R	36	−76	30	3.43
Postcenteral Gyrus	L	−22	−38	46	3.42
Middle Temporal Gyrus	R	60	−24	−10	3.41
Supramarginal Gyrus	R	48	−32	40	3.37
Superior Frontal Gyrus	R	26	28	54	3.36
Middle Cingulate Gyrus	R	8	−14	36	3.28
Middle Occipital Gyrus	L	−30	−74	22	3.25
Superior Parietal Lobule	L	−20	−64	44	3.24
Middle Temporal Gyrus	L	−62	−28	0	3.19
Lingual Gyrus	L	−8	−80	0	3.16

**Figure 5:**
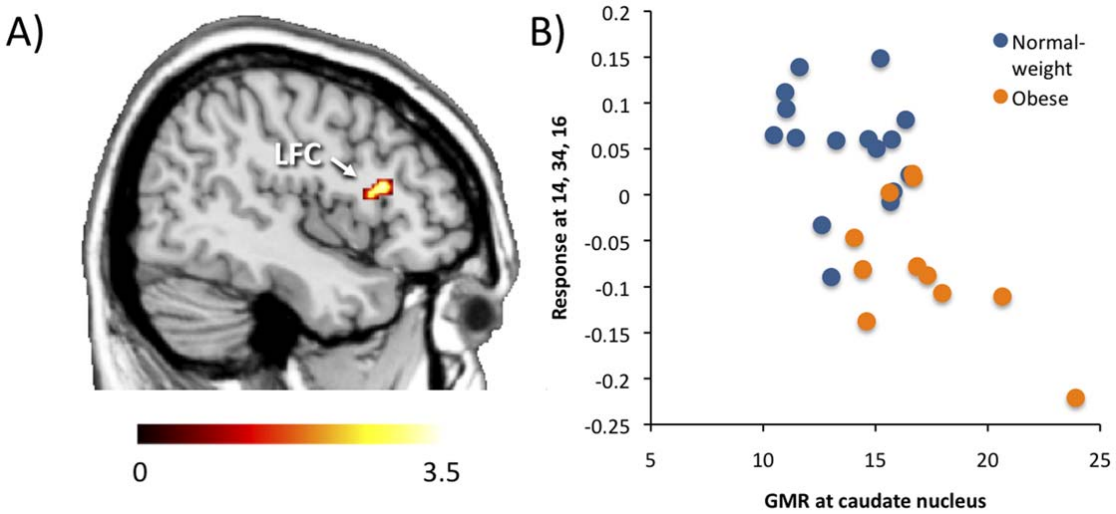
High glucose metabolic rate (GMR, µmol/100 g*min) in caudate nucleus during 2-[18F] FDG PET scan was negatively associated with responses to appetizing versus bland foods in right lateral frontal cortex (LFC) in the fMRI experiment. Panel A shows the region where the difference was observed, panel B shows a scatterplot of the GMRs and BOLD responses.

Finally, to examine if tonic hyperactivity of the caudate nucleus observed in the in [^18^F] FDG PET scan would predict abnormal anticipatory reward on fMRI, we first extracted subject-wise GMR values in the caudate nucleus from the parametric GMR images. Next, we used these values as a regressor in a second-level model comparing the BOLD responses to appetizing versus bland food in fMRI. This analysis showed that increased glucose metabolism in the caudate nucleus predicted smaller responses to appetizing versus bland food specifically in the right lateral frontal cortex (Figure 5). This finding is consistent with insufficient inhibitory control of subcortical reward systems by the frontal cortex.

### Psychophysiological Interactions

**Figure 6:**
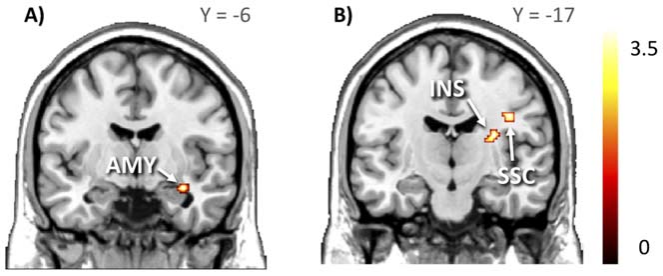
Effective connectivity. When viewing appetizing versus bland foods, the effective connectivity between right caudate nucleus and right amygdala (AMY), insula (INS) and somatosensory cortex (SSC) was greater in obese than in normal-weight subjects. The data are plotted at *p*<.005, uncorrected for visual inspection.

Having found evidence for a central role of caudate nucleus in mediating abnormal anticipatory reward in obesity, we next asked whether this brain region has abnormal functional task-related connectivity to other key brain regions, such as those of the limbic system. That is, we asked which brain regions would be central in modulating the anticipatory reward-related activity in the caudate nucleus while viewing appetizing versus bland foods. We used psychophysiological interactions to determine the functional connectivity of the caudate nucleus, using the voxel with the highest difference in glucose metabolism in the PET data as the center of the seed region. We found that obese subjects showed significantly stronger connectivity between right caudate nucleus and right basolateral amygdala (*X* = 33, *Y* = −5, *Z* = −16, *T* = 3.92, p<.005, unc.), primary somatosensory cortex (*X* = 39, *Y* = −13, *Z* = 32, *T* = 3.63, *p*<.005, unc.) and posterior insula (*X* = 30, *Y* = 14, *Z* = 18, *T* = 3.47, *p*<.005, unc.) than normal-weight subjects (Figure 6).

## Discussion

This study reveals the specific ways in which obesity modifies the responsiveness as well as functional connections of the reward circuit in the brain. Specifically, the results underline a central role for the dorsal caudate nucleus, a region promoting habitual learning and incentive motivation, in integrating various neural inputs in the process of anticipatory food reward. During hyperinsulinemia achieved with hyperinsulinemic euglycemic clamp, the dorsal caudate nucleus had higher basal glucose metabolism in obese subjects than in normal-weight subjects. The fMRI experiment showed that although the obese and normal-weight subjects gave similar self-reports to the pleasantness of the food stimuli, the stimuli elicited differential patterns of brain activation and changes in connectivity across the two groups. When appetizing and bland foods were contrasted with each other, the caudate nucleus showed greater response in the obese subjects. In contrast, obese subjects failed to activate cortical inhibitory regions, such as the dorsolateral and orbitofrontal cortices, in response to appetizing food; this phenomenon was also significantly correlated with higher basal glucose metabolism in the dorsal caudate nucleus. Finally, the very same region of the dorsal caudate nucleus that showed elevated glucose metabolism in obese versus normal-weight participants also showed increased connectivity with the amygdala and posterior insula in obese subjects while they were viewing appetizing versus bland foods. Importantly, these effects were observed under conditions where participants were not deliberately paying attention to the content of the stimulus pictures. Accordingly, the results suggest that *implicit reward processing* of visual cues for eating is modulated by obesity, which may explain why obese individuals have problems with restraining their eating upon seeing high-caloric foods. We must nevertheless note that is possible that the participants could have been engaged to some extent in explicit reward processing, even though the behavioural task was independent of the food pictures' reward value. Accordingly, future studies need to establish whether obese and normal-weight individuals could differ with respect to implicit versus explicit reward processing.

### Regional Differences in the Caudate Nucleus

Dorsal caudate nucleus has been implicated in habitual stimulus-response learning, motivation and conditioning, and imaging studies in humans suggest that it contributes to variety of functions related to reward signaling and addictions. Patients with drug addiction show lower baseline D_2_ receptor (D_2_R) density in the striatum, and blunted dopamine release following the administration of the drug of abuse [40]. Food consumption is also associated with dopamine release in the dorsal striatum in healthy subjects, and the amount of dopamine released is correlated positively with ratings of food pleasantness [12]. In fMRI experiments, activation of the caudate nucleus has been associated with self-reported craving for specific foods [8], and obese subjects have been found to show elevated striatal responses to food images [10]. Obese subjects have also lowered baseline striatal D_2_R density, and it has been proposed that this may reflect downregulation which compensates frequent transient dopamine increases due to perpetual overestimulation of the reward circuit by drug use or eating [11].

By using the hyperinsulinemic clamp, we simulated a situation where the body is in a satiated state in terms of insulin signaling. Although this approach does not completely simulate physiological satiety due to a lack of orosensory stimulation and release of hormones from the gut, placebo-controlled intravenous glucose has been shown to increase hormonal markers of satiety [41] and dopaminergic activity in the reward circuit in males [42]. We found that the dorsal striatum of the obese subjects remains hyperactive in comparison to normal-weight subjects during hyperinsulinemic clamp. As clamping maintains stable blood glucose levels, the elevated glucose metabolism in the obese subjects during clamp suggests the caudate nucleus of the obese subjects may contribute to food craving even when blood glucose concentration cannot decrease. Moreover, because of its involvement in implicit learning and habit formation, the caudate may contribute to processing of both implicit (peripheral) and explicit (visual, orosensory) satiety signals. These signals could subsequently lead to overeating even when the body would not require additional energy intake.

It has been established that in obese subjects, D_2_R availability in striatum is negatively associated with frontocortical glucose metabolism [43]. Our combined PET-fMRI data paralleled these findings. When glucose metabolism in caudate nucleus was used as a regressor for modeling the functional responses to appetizing versus bland foods in fMRI, we found a significant negative association with glucose metabolism in the caudate nucleus and prefrontal BOLD responses (Figure 5). Accordingly, failure to engage the prefrontal mechanisms contributing to inhibitory control and salience attribution could promote overeating by lowering the threshold for food-induced reward signaling in the caudate nucleus. However, it should also be noted that some prior studies [19] have reported *elevated* frontal responses to food pictures in obese versus normal-weight individuals. It is likely that these discrepancies across studies reflect task-dependent engagement of the frontal cortex: whereas our study involved implicit processing of briefly presented food cues, Rothemund and colleagues employed relatively long stimulus presentation with a memory task. It is thus possible that the obese individuals may fail to activate the cognitive control circuits particularly when they are not explicitly processing the food items they are viewing. Accordingly, this suggests that even ‘unseen’ or unattended food pictures in various advertisements could trigger powerful urges for eating in obese individuals.

### Effective Connectivity of Caudate Nucleus and Amygdala

The amygdala is involved in early stages of reward processing [44], and it shows consistent responses to visual presentations of foods [6], [22]. Individual differences in both reward drive [21] and body weight [10] are known to influence amygdala responses to visual presentations of foods. In the present study we also found that amygdala responses to foods were elevated in the obese subjects. Moreover, when effective connectivity patterns (PPIs) of caudate nucleus were inspected, we found that the connectivity of the caudate nucleus and the ipsilateral amygdala was elevated in the obese subjects. In general sense, these data accord with prior findings in normal-weight subjects showing that effective connectivity between amygdala and stratum is influenced by individual differences in self-reported desire to eat upon the sight of foods (‘external food sensitivity’) [22]. Nevertheless, whereas prior studies have found that particularly the ventral striatum is involved in reward anticipation [21] and that coupling between ventral striatum (nucleus accumbens) and amygdala is influenced by external food sensitivity [22], we found that obesity influenced the coupling between the amygdala and more dorsal parts of the caudate nucleus. The evidence regarding the role of dorsal striatum in reward processing is rather mixed, with some studies linking it to processing anticipatory [45] and others to consummatory [46] rewards. Nevertheless, the role of dorsal striatum in encoding action-outcome associations for potential rewards is much better established [47], [48]. Consequently, we propose that repeated exposures to palatable foods in obesity result in strong food stimulus-reward response associations and preferences, and implicitly engaged outcome evaluations regarding the potential rewards in obese individuals thus modulate the interconnectivity between the amygdala and the dorsal striatum upon sight of foods.

The interpretation of a significant PPI is that there is differential engagement of anatomical connections as a function of psychological context. Although the PPI cannot be used to reveal whether or not such connections exist, it is likely that the PPIs we observed reflect changes in the engagement of direct anatomical connections between the seed and target regions because such direct anatomical connections between the striatum and amygdala are supported by tracing studies in other primates [49], [50]. Nevertheless, the PPIs cannot be used to infer the directionality of the observed connectivity, hence we cannot say whether i) increased glucose metabolism in the caudate nucleus increases the connectivity between the caudate nucleus and amygdala or ii) increased inputs from amygdala increase the glucose metabolism in caudate nucleus.

Amygdala neurons facilitate reward seeking via their projections to the striatum [44]. Stimulation of the μ-opioid receptors in the striatum triggers overeating, but this can be blocked by inactivation of the amygdala [51], [52]. Accordingly, elevated amygdalo-striatal connectivity may lead to tonic increases in the activity of the caudate nucleus, which could be the critical mechanism explaining overeating in obesity. Taken together, amygdala might be involved in anticipated food reward by assigning emotional valence to appetizing food cues and influencing learned and compulsive eating patterns by enhanced connectivity with the dorsal caudate nucleus.

### Effective Connectivity of Caudate Nucleus and Insula

The PPI analyses revealed that the interconnectivity between dorsal striatum and posterior insula was elevated in the obese versus normal-weight subjects, whereas regional responses to appetizing versus bland foods in the *anterior* insula were smaller in the obese subjects. The anterior insula integrates autonomic and visceral signals into motivational and emotional functions, whereas the posterior insula is thought to underlie somatosensory, vestibular and motor integration as well as monitoring bodily states [53]. Recent work also points that somatosensory signaling in the insula may contribute significantly to addiction, particularly with urges to consume the drug of abuse (see review in ref. [53]). Prior PET and fMRI studies have linked insula to processing of pleasantness of external food cues [8], [9], [46], but peripheral signals such as leptin also influence insular response to seeing foods. In leptin-deficient adults, insular responses to appetizing foods are larger during leptin-deficiency rather than during leptin replacement [54]. Moreover, in obese subjects with leptin deficiency, leptin replacement dampens insular responses to viewing appetizing foods [55]. As the insula processes both internal (i.e. hormonal) and external (i.e. visual) food-related cues [56], disruptions in this integration of internal and external cues may render obese subjects more prone to overeating upon the sight of foods due to the elevated connectivity from insula and dorsal striatum. Since posterior insula is involved in monitoring bodily states, enhanced connectivity between posterior insula and dorsal caudate nucleus might imply that recalled representations of post-prandial somatic states by the insula might potentially reinforce feeding behaviors through incentive learning subserved by the dorsal caudate nucleus [18]. Consistent with this notion, the caudate nucleus also showed higher task-related connectivity with somatosensory cortex in obesity, confirming that mere visual cues of foods might trigger somatic sensations associated with eating. These sensations may further promote feeding even in the absence of physiological hunger signals [15]. Nevertheless, it must be noted that some prior studies have found *elevated* anterior insular responses to expected and consummatory food-related rewards in obese rather than in lean individuals [10], [57]. Although we have no clear explanation to these discrepant findings, it is possible that they may reflect differences in the obese subject populations involved in the studies, such as eating history and habits as well as genetic and hormonal factors.

### Limitations and future directions

One obvious limitation of the present study was that despite a large sample size (*n* = 35) the between-group comparisons for fMRI data were not significant when corrected for multiple comparisons. Although the between-group differences were observed in predicted regions, some caution should be warranted when interpreting the findings. Furthermore, it must be stressed that we were not able to fully delineate the exact psychological mechanism that results in elevated brain responses to food pictures in obese individuals. Although we acquired ratings of the perceived pleasantness (‘liking’) of the foods, these were similar across obese and normal-weight individuals. Accordingly, elevated liking of appetizing foods in obesity is unlikely to contribute to the differences in brain responses. However, it could be speculated that food *craving* rather than liking could be the key factor that modulates brain responses to food pictures in obesity. In support of this hypothesis, it has been shown that although obese and normal-weight individuals ‘like’ foods similarly, stress-induced food craving is much higher in obese individuals [58]. In future functional imaging studies, it would thus be imperative to disentangle the ‘craving’ and ‘liking’ responses to foods in obese versus normal-weight individuals. Furthermore, given that craving responses are mediated by the dopaminergic link of the reward circuit, [24], it would be imperative to conduct combined neurotransmitter-PET-fMRI studies in which one could test whether, for example, striatal dopamine availability in obese vs. lean individuals predicts reward circuit's responses to external stimulation with foods.

### Conclusion

We show that obesity is associated with elevated glucose metabolism of the caudate nucleus, as well as modified regional responses and altered connectivity of the reward circuit when seeing appetizing versus bland foods. These data parallel with the findings on altered brain functioning in addictive disorders, and support the view that obesity may share a common neural substrate with addictions [2], [59]. Specifically, enhanced sensitivity to external food cues in obesity may involve abnormal stimulus-response learning and incentive motivation subserved by the dorsal caudate nucleus, which in turn may be due to abnormally high input from the amygdala and posterior insula and dysfunctional inhibitory control by the frontal cortical regions. These functional changes in the responsiveness and interconnectivity of the reward circuit and cognitive control systems could be a critical mechanism that explains overeating in obesity.
